# Spatial scan statistics for matched case-control data

**DOI:** 10.1371/journal.pone.0221225

**Published:** 2019-08-16

**Authors:** Inkyung Jung

**Affiliations:** Division of Biostatistics, Department of Biomedical Systems Informatics, Yonsei University College of Medicine, Seoul, Korea; Johns Hopkins Bloomberg School of Public Health, UNITED STATES

## Abstract

Spatial scan statistics are widely used for cluster detection analysis in geographical disease surveillance. While this method has been developed for various types of data such as binary, count, and continuous data, spatial scan statistics for matched case-control data, which often arise in spatial epidemiology, have not been considered. We propose spatial scan statistics for matched case-control data. The proposed test statistics consider the correlations between matched pairs. We evaluate the statistical power and cluster detection accuracy of the proposed methods through simulations compared to the Bernoulli-based method. We illustrate the proposed methods using a real data example. The simulation study clearly revealed that the proposed methods had higher power and higher accuracy for detecting spatial clusters for matched case-control data than the Bernoulli-based spatial scan statistic. The cluster detection result of the real data example also appeared to reflect a higher power of the proposed methods. The proposed methods are very useful for spatial cluster detection for matched case-control data.

## Introduction

Spatial cluster detection is an important problem in spatial epidemiology. Among the various statistical methods available, the spatial scan statistic [1] is one of the most widely used methods. Application of this method is not limited to geographical disease surveillance, but to various areas, including criminology [2,3], entomology [4], and urban planning [5,6]. The spatial scan statistic is defined as the maximum of likelihood ratio test statistics over a collection of scanning windows. Numerous scanning windows are constructed on an entire study region and each is a candidate for the most likely cluster. The likelihood ratio test statistic for comparing the inside versus the outside of a window is formulated based on the data type to be analyzed. Different probability models for the spatial scan statistic have been proposed and extensively used such as Poisson [1], Bernoulli [1], ordinal [7], multinomial [8], normal [9], and exponential [10]. The freely available software SaTScan [11] can be used for the probability models mentioned above.

In epidemiology, one frequently used retrospective observational study design is a case-control study, in which cases with an outcome of interest are identified and a comparable control group is sampled. Further, controls matching each case can be selected to control for confounding variables. In some studies, cases and controls could be matched by their location as well. When we are interested in spatial variation between cases and controls, however, we should not consider their location information as a confounding variable. Matched case-control data require a specific form of analysis to consider dependency in responses within a matched pair. However, there is no spatial scan statistic for matched case-control data. Some studies have used the Bernoulli-based spatial scan statistic [12, 13]. Because the method was developed for independent binary outcome data, it may be inappropriate to apply the Bernoulli-based method to matched case-control data.

In this paper, we propose two spatial scan statistics for matched case-control data. The test statistics are constructed based on McNemar’s test statistic and Wald type test statistic for an odds ratio. In the next section, we briefly review the Bernoulli-based spatial scan statistic and then present the proposed methods. Through a simulation study, we evaluated the performance of the proposed methods compared to that of the Bernoulli-based method in terms of statistical power and detection accuracy. We illustrate the proposed methods using a real data example of male lung cancer cases with matched controls in Seoul, Korea. We provide conclusions and discussion in the final section.

## Methods

### Spatial scan statistic for binary data

For binary outcome data such as cases and non-cases of certain diseases, we can use the Bernoulli-based spatial scan statistic. The null and alternative hypotheses are written as

$$H_{0}:p=q\;for\;all\;z\in Z\;vs.\;H_{a}:p>q\;for\;some\;z\in Z$$ (1)

where *p* and *q* are the probability of being a case inside and outside the scanning window *z*, respectively, and Z denotes the collection of all scanning windows. Scanning windows are constructed at every location with varying sizes by including the nearest neighbor one by one, up to certain limit. Usually 50% of total population is set as the maximum value of scanning window size. Given window *z*, the test statistic is expressed as

$$\begin{array}{c} LR\left(z\right)=\frac{{\left(\frac{c_{z}}{n_{z}}\right)}^{c_{z}}{\left(\frac{n_{z}-c_{z}}{n_{z}}\right)}^{n_{z}-c_{z}}{\left(\frac{C-c_{z}}{N-n_{z}}\right)}^{C-c_{z}}{\left(\frac{N-n_{z}-\left(C-c_{z}\right)}{N-n_{z}}\right)}^{N-n_{z}-\left(C-c_{z}\right)}}{{\left(\frac{C}{N}\right)}^{C}{\left(\frac{N-C}{N}\right)}^{N-C}}\\ ∙I(\frac{c_{z}}{n_{z}}>\frac{C-c_{z}}{N-n_{z}}) \end{array}$$

where *c*_*z*_ and *n*_*z*_ denote the number of cases and observations within *z*, respectively, and *C* and *N* are the total number of cases and observations over the whole study area, respectively. I() is an indicator function to indicate the high or low rate. To search for a cluster with a low rate (*H*_*a*_: *p*<*q*), the inequality sign in the indicator function should be in the opposite direction. If we want to search for clusters with either high or low rates, the indicator function is eliminated. Because the denominator in the above formula does not depend on *z*, the term *(C/N)*^*C*^*((N-C)/N)*^*N-C*^ can be eliminated.

The scanning window associated with the maximum value of *LR*(*z*) is defined as the most likely cluster. The Monte Carlo hypothesis testing is the standard method for obtaining a p-value for the most likely cluster. In addition to the most likely cluster, we often report secondary clusters with high values of *LR*(*z*). The p-values of secondary clusters are typically obtained in the same manner. The Bernoulli-based spatial scan statistic is available on SaTScan.

### Spatial scan statistics for matched case-control data

When we have binary outcome data from a matched case-control study, it may not be appropriate to use the Bernoulli-based spatial scan statistic described above. The Bernoulli-based scan statistic is used for independent observations. The case and control within a matched pair are not independent. In addition, the hypotheses should be expressed in a different manner from (1) because the probability of being a case is not meaningful in matched case-control data.

Suppose that we have *n* matched pairs, each of which was formed from one case and one control. Given window *z*, there are four possible statuses for each case-control pair with respect to whether they belong to *z* or not, as shown in Table 1. Both the case and control can belong to z, only one can, or neither could. Table 1 shows the probability (data) structure for the four possible states. For example, *π*_11_(*n*_11_) denote the probability (number of pairs) of belonging to window *z* for both the case and control. Then, we can express the hypotheses to search for clusters with high rates as follows.

$$H_{0}:\pi_{1+}=\pi_{+1}\;for\;all\;z\in Z\;vs.\;H_{a}:\pi_{1+}>\pi_{+1}\;for\;some\;z\in Z$$ (2)

$$\mathrm{or}\;H_{0}:\pi_{10}=\pi_{01}\;for\;all\;z\in Z\;vs.\;H_{a}:\pi_{10}>\pi_{01}\;for\;some\;z\in Z.$$ (3)

The situations satisfying the null hypotheses in (2) and (3) are referred to as marginal homogeneity and symmetry, respectively. Equivalently, we may write the hypotheses in (3) using an odds ratio (OR = *π*_10_/*π*_01_) as

$$H_{0}:\pi_{10}/\pi_{01}=1\;for\;all\;z\in Z\;vs.\;H_{a}:\pi_{10}/\pi_{01}>1\;for\;some\;z\in Z.$$ (4)

Here, we propose utilizing McNemar’s test statistic and Wald-type test statistic for the OR to define spatial scan statistics for matched case-control data. We define the first test statistic given *z* as

$$T_{z}^{\left(1\right)}=\frac{{\left(n_{10}-n_{01}\right)}^{2}}{n_{10}+n_{01}}I(n_{10}>n_{01})$$

and the second test statistic given *z* as

$$T_{z}^{\left(2\right)}=\frac{{\left\{log(n_{10}/n_{01})\right\}}^{2}}{1/n_{10}+1/n_{01}}I(n_{10}>n_{01}).$$

The area with the maximum value of $T_{z}^{\left(1\right)}$ or $T_{z}^{\left(2\right)}$ over *z*∈*Z* becomes the most likely cluster. $T_{z}^{\left(1\right)}$ is simply the McNemar’s test statistic. $T_{z}^{\left(2\right)}$ is the squared Wald test statistic for log OR. log(*n*_10_/*n*_01_) is the conditional maximum likelihood (ML) estimate of log OR (log(*π*_10_/*π*_01_)), and $\sqrt{1/n_{10}+1/n_{01}}$ is its standard error estimate. To search for clusters with low rates, we use the indicator function with the reversed inequality sign.

**Table 1:** Probability (data) structure for the matched case-control data with respect to belonging to window z (in) or not (out).

For a given *z*		Control		
		in	out	
Case	in	*π*_11_ (*n*_11_)	*π*_10_ (*n*_10_)	*π*_1+_ (*n*_1+_)
	out	*π*_01_ (*n*_01_)	*π*_00_ (*n*_00_)	*π*_0+_ (*n*_0+_)
		*π*_+1_ (*n*_+1_)	*π*_+0_ (*n*_+0_)	1 (*n*)

Although $T_{z}^{\left(1\right)}$ and $T_{z}^{\left(2\right)}$ are known to follow a chi-square distribution asymptotically, we do not know the null distributions of $T^{\left(1\right)}=\max_{z\in Z}T_{z}^{\left(1\right)}$ or $T^{\left(2\right)}=\max_{z\in Z}T_{z}^{\left(2\right)}$. As for the standard spatial scan statistics, we use Monte Carlo hypothesis testing procedure for the statistical inference of the clusters detected using the proposed methods. Under the null hypothesis, we generate a large number of data sets by randomly permuting the locations of observations with matching ids fixed. Then, we calculate the maximum values of test statistics for each data set. In that way, we obtain empirical null distributions of the proposed test statistics. The Monte Carlo-based p-value for the detected cluster is the rank of the maximum value of the test statistics from the real data set among all data sets divided by the number of all data sets.

## Results

### Simulation study

**Fig 1:**
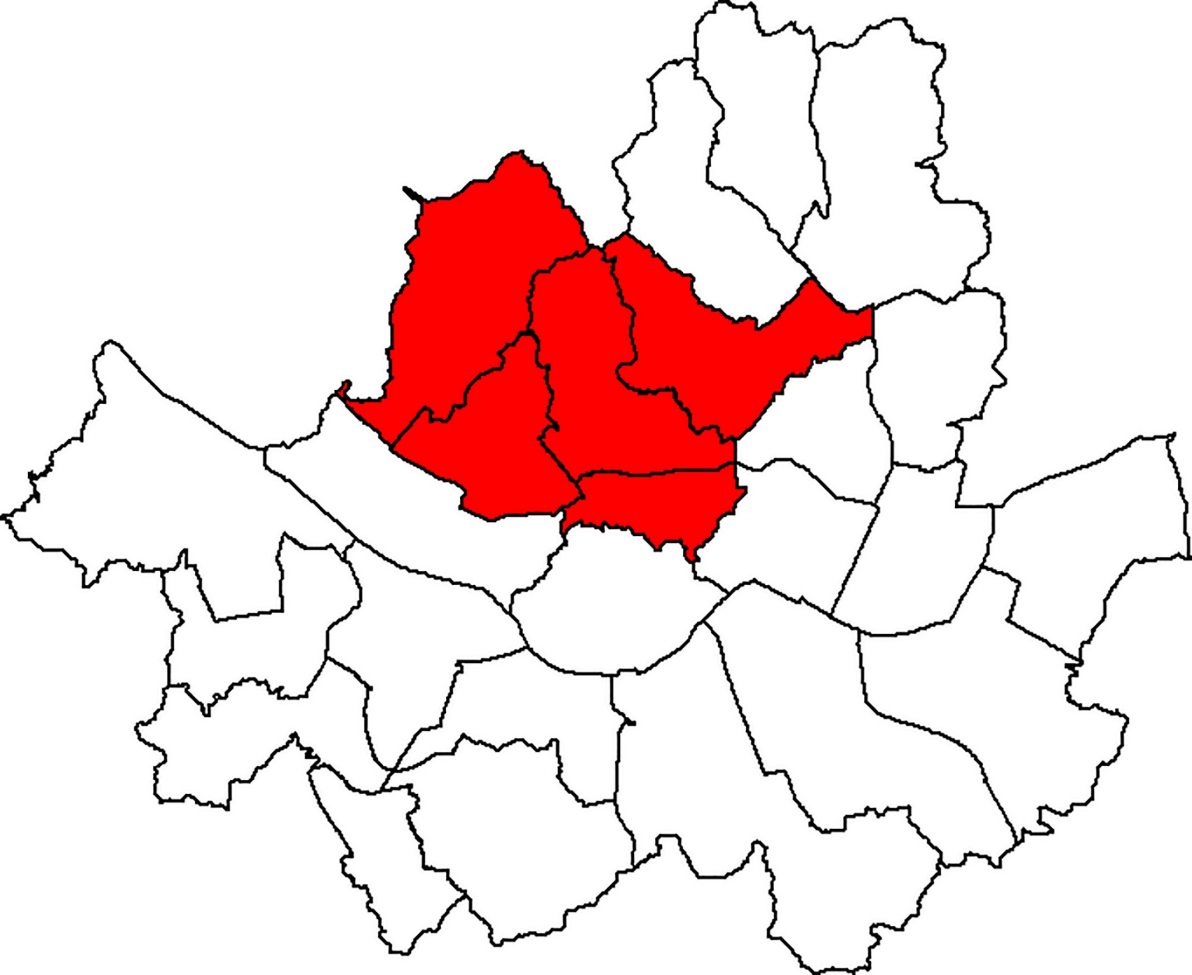
A true cluster created for the simulation study.

We conducted a simulation study to evaluate the performance of the proposed methods. We used the area of Seoul, the capital city of South Korea, as the whole study region. Seoul is composed of 25 districts. We created a true cluster consisting of 5 districts in the northwest area as shown in Fig 1. The cluster include “Jongno-gu” district with 4 nearest neighbors. We set the total number of matched pairs to 100, 200, and 400. When searching for clusters, we set the maximum scanning window size to 50% of total number of matched pairs. We considered 5 different scenarios for the probabilities of *π*_*ab*_ as shown in Table 2. The first scenario was included to evaluate whether the proposed methods adequately control the type I error rate. Across the 4 scenarios except for the first one, the odds ratios (ORs) (*π*_10_/*π*_01_) are different, while the unconditional marginal ORs (*π*_1+_*π*_+0_/ *π*_0+_*π*_+1_) are the same. The unconditional marginal OR refers to the OR as if we deal with the data from an unmatched case-control study. The 4 scenarios provide different information on the magnitude of risk for the cluster. However, the unconditional marginal OR cannot account for this.

**Table 2:** Five different scenarios assumed for the probability structure in the simulation study.

(*π*_11_, *π*_10_, *π*_01_, *π*_00_)	*π*_10_/*π*_01_	*π*_1+_*π*_+0_/ *π*_0+_*π*_+1_
(0.25, 0.25, 0.25, 0.25)	1	1
(0.05, 0.25, 0.15, 0.55)	1.67	1.71
(0.10, 0.20, 0.10, 0.60)	2	1.71
(0.15, 0.15, 0.05, 0.65)	3	1.71
(0.18, 0.12, 0.02, 0.68)	6	1.71

At each scenario, (*n*_11_,*n*_10_,*n*_01_,*n*_00_) were first generated from a multinomial distribution with index *n* and parameter (*π*_11_,*π*_10_,*π*_01_,*π*_00_). We set the same identification number to each pair of case and control to indicate matching from 1 to *n*. Then, each of cases and controls of the first *n*_11_ pairs were randomly assigned to one of 5 districts of the true cluster. Cases and controls of *n*_10_ pairs were randomly assigned to districts inside and outside the true cluster, respectively. Similarly, cases and controls of *n*_01_ pairs were randomly assigned to districts outside and inside the true cluster, respectively. Both the cases and controls of *n*_00_ pairs were randomly assigned to districts outside the true cluster.

We compared the performance of the two proposed methods to that of the Bernoulli-based method. We estimated the power, sensitivity, and positive predicted value (PPV) from 1000 replications. Power was estimated as the number of rejected data sets out of 1000. For the first scenario (OR = 1), power is the type I error rate. Because power cannot show the accuracy of detected clusters, sensitivity and PPV were used to evaluate how accurately the methods can detect clusters. Sensitivity was defined as the proportion of districts detected correctly among the districts in the true cluster and PPV as the proportion of districts detected correctly among the districts in the detected cluster. These are commonly used when reporting simulation results in studies on spatial scan statistics [7–10, 14–17]. Larger values of sensitivity and PPV indicate higher accuracy of detected clusters. Sensitivity and PPV were estimated as the average among the rejected samples.

Both the proposed methods and the Bernoulli-based method controlled the type I error rate less than the nominal level of 0.05. Estimated type I error rates for *T*^(1)^ and *T*^(2)^ were 0.030, 0.036, and 0.042, and 0.028, 0.034, and 0.039 for the number of matched pairs = 100, 200, and 400, respectively. The Bernoulli model had the type I error rates of 0.034, 0.026, and 0.041.

Tables 3–5 show the simulation results listing the estimated power, sensitivity, and PPV with the number of matched pairs = 100, 200, and 400, respectively. Overall, the two proposed methods showed higher power, sensitivity, and PPV than the Bernoulli-based method. Moreover, the power of the proposed methods increased as the OR increased. However, the Bernoulli-based method showed similar performances across different scenarios because we assumed the same marginal OR.

**Table 3:** Estimated power, sensitivity, and PPV with the number of matched pairs = 100 (highest value across three methods is shown in bold).

(*π*_11_, *π*_10_, *π*_01_, *π*_00_)		*T*^(1)^	*T*^(2)^	Bernoulli-based
(0.05, 0.25, 0.15, 0.55)	Power	0.153	**0.156**	0.113
	Sensitivity	0.799	**0.842**	0.720
	PPV	0.623	0.603	**0.683**
(0.10, 0.20, 0.10, 0.60)	Power	**0.196**	0.190	0.110
	Sensitivity	0.820	**0.854**	0.691
	PPV	**0.689**	0.633	0.676
(0.15, 0.15, 0.05, 0.65)	Power	**0.249**	0.187	0.085
	Sensitivity	0.865	**0.882**	0.664
	PPV	**0.760**	0.605	0.596
(0.18, 0.12, 0.02, 0.68)	Power	**0.349**	0.207	0.078
	Sensitivity	**0.908**	0.886	0.562
	PPV	**0.793**	0.567	0.586

**Table 4:** Estimated power, sensitivity, and PPV with the number of matched pairs = 200 (highest value across three methods is shown in bold).

(*π*_11_, *π*_10_, *π*_01_, *π*_00_)		*T*^(1)^	*T*^(2)^	Bernoulli-based
(0.05, 0.25, 0.15, 0.55)	Power	0.289	**0.303**	0.184
	Sensitivity	0.845	**0.863**	0.837
	PPV	0.732	0.715	**0.781**
(0.10, 0.20, 0.10, 0.60)	Power	**0.375**	0.372	0.232
	Sensitivity	0.864	**0.888**	0.799
	PPV	**0.764**	0.744	0.734
(0.15, 0.15, 0.05, 0.65)	Power	**0.563**	0.548	0.210
	Sensitivity	0.923	**0.930**	0.766
	PPV	**0.865**	0.817	0.684
(0.18, 0.12, 0.02, 0.68)	Power	**0.811**	0.678	0.158
	Sensitivity	**0.958**	0.925	0.747
	PPV	**0.921**	0.803	0.683

**Table 5:** Estimated power, sensitivity, and PPV with the number of matched pairs = 400 (highest value across three methods is shown in bold).

(*π*_11_, *π*_10_, *π*_01_, *π*_00_)		*T*^(1)^	*T*^(2)^	Bernoulli-based
(0.05, 0.25, 0.15, 0.55)	Power	0.585	**0.598**	0.586
	Sensitivity	0.886	**0.897**	0.882
	PPV	**0.849**	0.811	**0.849**
(0.10, 0.20, 0.10, 0.60)	Power	0.737	**0.743**	0.648
	Sensitivity	0.923	**0.928**	0.878
	PPV	**0.859**	0.848	0.821
(0.15, 0.15, 0.05, 0.65)	Power	**0.920**	0.914	0.689
	Sensitivity	0.966	**0.969**	0.865
	PPV	**0.944**	0.927	0.837
(0.18, 0.12, 0.02, 0.68)	Power	**0.996**	0.995	0.684
	Sensitivity	**0.993**	0.983	0.842
	PPV	**0.981**	0.943	0.820

We observed some consistent patterns in the results using the two proposed methods. *T*^(1)^ showed higher power than *T*^(2)^ except for the case of *π*_10_/*π*_01_ = 1.67. PPV was always higher in results from *T*^(1)^ than from *T*^(2)^. *T*^(2)^ showed higher sensitivity than *T*^(1)^ except for *π*_10_/*π*_01_ = 6. Although the difference in performance between the two proposed methods was marginal, *T*^(1)^ appeared to perform slightly better under the scenarios we assumed.

### Real data example

We used national health insurance service national sample cohort data, which is a randomly selected sample from population-based cohort data set containing insurance eligibility, medical treatments, medical care institutions, and general health examinations [18]. The data comprise approximately 2.2% of total eligible Korean population. Residential area information was available at the district level. From the sample cohort data, we identified 173 male cases diagnosed with lung cancer at general health examination for the year of 2013 in Seoul. Next, we randomly selected 173 controls with age-group matched to each case.

**Fig 2:**
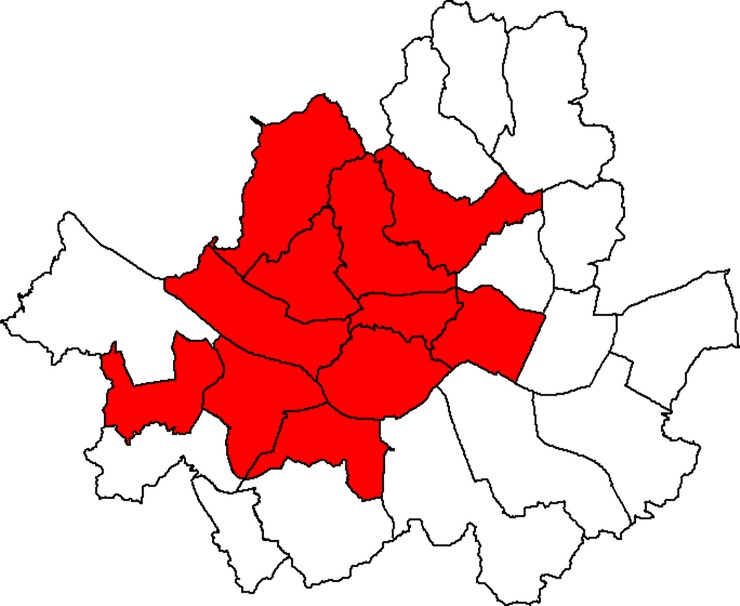
The most likely cluster detected by the two proposed methods.

We applied the two proposed methods and the Bernoulli-based method to the data. The Bernoulli-based method revealed no significant clusters, while the two proposed methods detected a significant cluster consisting of 11 districts in the midwest area of Seoul, with a p-value equal to 0.047 and 0.044, respectively (Fig 2). The observed counts for the matched pairs with respect to whether they were inside or outside the cluster were *n*_11_ = 19, *n*_10_ = 53, *n*_01_ = 25, and *n*_00_ = 76. The conditional ML estimate for the OR was 2.12. The cluster is the region with higher odds of male lung cancer than the remaining region, adjusting for age-group. In fact, the same area was the most likely cluster when the Bernoulli-based method was used, but the statistical significance was not obtained (p-value = 0.09). As shown in the simulation study, the result may reflect that the proposed methods have higher power than the Bernoulli-based spatial scan statistic for matched case-control data.

## Discussion and conclusion

We have proposed two spatial scan statistics for matched case-control data in this paper. The methods are based on McNemar’s test statistic and Wald-type test statistic for the OR. Therefore, we accounted for the correlation in responses within a matched pair. If we use the Bernoulli-based spatial scan statistic for matched case-control data, we ignore the correlation, and the cluster detection test results will suffer from low power.

The simulation study clearly revealed that the proposed methods had higher power and higher accuracy for detecting spatial clusters for matched case-control data than the Bernoulli-based spatial scan statistic. The cluster detection result for the male lung cancer data also appeared to reflect a higher power of the proposed methods. The method based on McNemar’s test statistic appeared to perform slightly better than the other proposed method, although the difference was marginal.

We have considered searching for clusters with high rates expressed with OR>1. We might be interested in clusters with low rates, i.e., OR<1. Because an OR is symmetric about 1, the reciprocal of an OR represents the same strength of association in opposite direction. Searching for clusters with OR<1 for cases relative to controls is identical to searching clusters with OR>1 for controls relative to cases. We can see this symmetry in the test statistics as well. Both the test statistic values of $T_{z}^{\left(1\right)}$ and $T_{z}^{\left(2\right)}$ remain the same even if *n*_10_ and *n*_01_ are switched except the identity function part. We can use the proposed methods to search for clusters with low rates by only switching the direction of inequality sign in the identity function.

Here, we focused on the simplest situation of 1:1 matching, where one control was matched to each case. When multiple controls were matched to a single case (M:1 matching), we can still use the Wald-type test statistic for the regression coefficient from conditional logistic regression modeling. We may rewrite the hypotheses in (4) and test statistic $T_{z}^{\left(2\right)}$ as *H*_0_:*β* = 0 for all *z*∈*Z vs*. *H*_*a*_:*β*>1 for some *z*∈*Z* in a conditional logistic regression model as follows.

$$logit(P(Y_{it}=1))=\alpha_{i}+\beta x_{it},i=1,\ldots ,n,\;t=1,2$$ (5)

where *x*_*it*_ = 1 if subject *t* (*t* = 1 if case and *t* = 2 if control) in matched pair *i* belongs to *z* and *x*_*it*_ = 0 otherwise, and

$$T_{z}^{\left(2\right)}=\frac{{\hat{\beta }}^{2}}{1/n_{10}+1/n_{01}}I(\hat{\beta }>0).$$

For 1:1 matched data, the OR (*π*_10_/*π*_01_) is identical to exp(*β*). The conditional logistic model (5) can be extended to *M*:1 matched data. We only need to modify the model using *t* = 1,…,*M*+1 to indicate one case and *M* controls. We can estimate *β* and the estimate’s standard error based on the conditional ML method. Then, the Wald-type test statistic can be constructed in the same manner. However, further evaluation in a simulation study is warranted to evaluate the method.

We only considered circular windows. Other shapes of scanning windows such as elliptic or irregular windows have been extensively studied [17, 19–24]. It would be interesting to evaluate the proposed methods using other shapes of windows. In addition to the proposed test statistics in this paper, it may be possible to use other types of test statistics for matched case-control data.

In conclusion, the proposed methods are very useful for spatial cluster detection for matched case-control data.

## Supporting information

S1 File A zip file including the lung cancer data set and a sample R code. (ZIP)
