# Supplementary material for: Spatial scan statistics for matched case-control data
Source: PLoS One. 2019 Aug 16;14(8):e0221225. doi: 10.1371/journal.pone.0221225 (PMC6697355; doi:10.1371/journal.pone.0221225)
Supplement: S1 File — (ZIP) [file pone.0221225.s001.zip › Data and code.docx]

Data and code explanation

- csctl_male_lung.csv: matched case-control data of lung cancer in Seoul with location information

id: matching id

locid: location (district) id

csctl: whether the subject is a case (=1) or a control (=0)

- s_centroid.csv: x and y coordinates of centroid for each district in Seoul (25 districts in total)

- scanningw.csv: indicators of scanning windows

- Male_lungcancer.R: R codes to run proposed methods for this particular data set
